# Properties of Ceramic Coating on Heating Surface of Waste Incineration Boiler Prepared by Slurry Method

**DOI:** 10.3390/ma15134574

**Published:** 2022-06-29

**Authors:** Zengzhi Wei, Lijun Wu, Xingyuan Liang

**Affiliations:** School of Mechanical Engineering, Tongji University, Shanghai 201804, China; 2032771@tongji.edu.cn (Z.W.); 2032772@tongji.edu.cn (X.L.)

**Keywords:** waste incineration boiler, ceramic coating, antiscaling, high temperature contact angle, mechanical properties

## Abstract

In order to alleviate the problem of high-temperature fly ash corrosion and slag on the heating surface of a high-parameter waste incinerator, a ceramic coating material that can be prepared in situ on the heating surface by the slurry method was studied. The ceramic coating can be formed by sintering at a lower temperature of 750 °C. Its surface and profile are very dense, and the porosity is less than 1%. The mechanical properties test results show that the ceramic coating can withstand 60 cycles of water-cooled thermal shock at 700 °C, and the bonding strength is 25.14 ± 2.21 MPa. It will not fall off in a large area when subjected to pressure load, and it has a certain degree of processable plasticity. High-temperature wettability experiments show that the ceramic coating has lower liquid-bridge force, smaller adhesion area, and shorter fouling cycle for molten corrosive fouling, and potential self-cleaning properties. Its practical mechanical properties make the coating valuable for production applications and meet expectations, and excellent antifouling properties to reduce average fouling thermal resistance and corrosion.

## 1. Introduction

With the increasingly prominent contradiction between environmental pollution and energy supply and demand, waste incineration power plants have become a realistic choice for large cities. According to relevant reports, there are more than 2100 waste incineration power plants globally with an incineration capacity of 230 million tons, reducing the volume of refuse disposed of in dumps by 90% [1,2]. However, with the increase in power generation efficiency requirements, the problem of high-temperature corrosion and deposition on the heating surface has become more and more serious, so has become a key factor restricting the production efficiency and safety of the systems [3,4]. Due to the complexity and heterogeneity of solid waste, it usually contains high concentrations of various chlorine-containing compounds and alkali metals. These substances form many low-melting-point liquid corrosive substances that adhere to the incinerator wall, resulting in waste incinerators experiencing more severe scaling corrosion problems than most coal-fired boilers [5,6].

To solve these problems, many coating technologies have been developed for the treatment of boiler heating surfaces. Research shows that the current coating technology to deposit a layer of high-performance alloy on the heating surface and protect the inner layer substrate by sacrificing the outer layer alloy mainly include thermal spraying [7], laser cladding [8], and welding [9]. However, these methods cannot alleviate the aggravation of high-temperature corrosion and are only used as substitutes for corrosion. These coating technologies essentially thicken the heating surface to prolong the service life, thereby prolonging the time for the wall to be thinned to a critical thickness. In addition, because high-performance alloys are costly and limited by economic benefits, they are difficult to apply in small- and medium-sized power plants. Ceramic materials have attracted the attention of researchers because of their excellent high-temperature corrosion resistance [10], lower material cost [11], and more straightforward preparation process; they have become a very promising alternative alloy material. Because this research has just started, the sintering temperature of ceramic coatings is usually above 1000 °C [10,11], and the sudden change in the physical properties between a ceramic coating and metal substrate leads to low bonding strength and poor comprehensive mechanical properties, so they cannot be applied on a large scale.

In an incineration boiler, the corrosion and scaling of the heating surface are usually synergistic. Scholars have conducted various studies on feasible methods to eliminate slagging. Some scholars have suggested using additives, which can be physically absorbed or chemically reacted with sodium in the gas phase [12,13]. Some scholars have also suggested that fuels with a low fouling tendency should be mixed with waste to burn, thereby increasing the melting point of ash and reducing liquid molten fouling [14]. The current antiscaling methods struggle to meet the requirements in applications. There is no study reported in the literature of slagging resistance of new coating materials.

Based on practical applications, to alleviate the scaling and corrosion problems of boiler heating surfaces, a ceramic coating that can be sintered at low temperature was prepared by the slurry method, with mica powder as the main filler and sodium silicate water glass as the binder. Through the analysis of the macro- and micromorphology of the ceramic coating, the forming quality of the ceramic coating was discussed, and further mechanical properties tests were carried out to judge the effects of its practical application. According to the unique characteristics of the prepared ceramic coating, the antiscaling mechanism under a high-temperature environment was analyzed. The results of this study provide a reference for the subsequent development of similar ceramic coatings.

## 2. Materials and Methods

### 2.1. Coating Slurry Preparation

The raw powder used to prepare the ceramic coating consisted of mica powder (mean particle size: 1 μm), copper powder, graphite powder, and chromium oxide powder (purity: >99%; mean particle size: 500 nm). Sodium silicate water glass with a modulus of 2.5 was used as the coating binder.

In general, an ultrafine powder has a relatively high surface activity and requires low energy for surface reaction, and the coating can be densified below the common reaction or sintering temperature. However, an ultrafine powder tends to agglomerate into large solid masses, and the dispersant and dispersing methods were used in combination to solve this problem. Among them, the dispersant and defoamer were sodium polyacrylate and triglyceride, respectively, which were dispersed by stirring first and then ultrasonic treatment. The initial slurry was prepared and mixed to obtain the composition shown in Table 1.

### 2.2. Coating Preparation

The material commonly used in high-performance boilers, SUS304 steel, was used as the substrate in the present study. After sandblasting the substrate, the steel substrate surface was coated with the ceramic slurry by a high-volume, low-pressure (HVLP; WS4000D, SATHN Co., Ltd., Anhui, China) spraying method to obtain a homogeneous film. The intake pressure of the HVLP spray gun was 0.3 MPa; the distance between the spray nozzle and the substrate was 20–30 cm. After spraying, the coating was naturally dried at a room temperature of about 25 °C for 24 h to complete the geopolymerization process. Then, it was sintered in a muffle furnace.

The heating surface of the start-up boiler of waste incineration power plants can reach 700 °C, and the flue gas temperature can reach more than 900 °C. Therefore, the coating can be sintered and formed by using the high temperature in the boiler. To simulate the actual situation where the coating is sintered in the boiler, the muffle furnace was set to reach 750 °C at a heating rate of 1 °C/min and then maintained for 10 min, after which the specimen was cooled naturally and then removed from the furnace.

### 2.3. Mechanical Properties Evaluation

#### 2.3.1. Evaluation of Thermal Shock Resistance

A water-cooled thermal shock test was used to evaluate the ability of the coating to withstand extremely frequent and rapid temperature changes within the boiler. We put the specimen in muffle furnace subjected to high temperature for 5 min, took it out, quickly put it into the normal temperature water at 23 °C, and then put it into the muffle furnace for circulation until the coating rupture area reached 5%. Three groups of experimental temperatures (500, 600, and 700 °C) were set, and the average value of the three tests was taken for the rupture area.

#### 2.3.2. Bonding Performance Test

According to the ASTM C1624 test standard, a quantitative scratch adhesion test was conducted on the coating with material surface performance tester (MFT 4000, MTS Industrial System Co., Ltd., Shenzhen, China) to evaluate its practical adhesion performance. The load was 50 N, the loading time was 30 s, and the loading distance was 3 mm. The fundamental adhesion between the coating and substrate was evaluated according to ASTM C663 01 using a mechanical testing machine (SANS, CMT5105, MTS Industrial System Co., Ltd**.**, Shenzhen, China), which measures the force required to peel the specified area of coating from the plate.

### 2.4. High Temperature Wettability Test

The wettability of the liquid on a solid surface can be characterized by the static contact angle *θ*, which is the angle between the gas–liquid and solid–liquid interfacial tension at the junction of the solid, liquid, and gas. The greater the *θ*, the lower the wettability.

**Figure 1:**
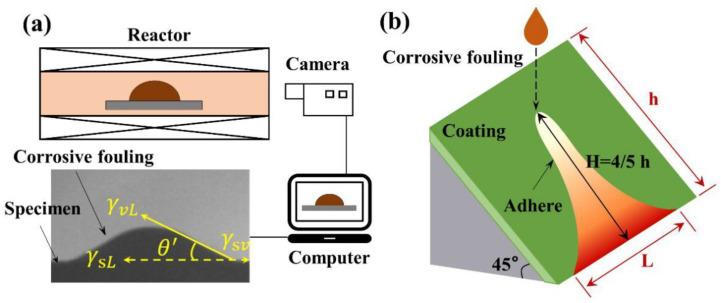
Schematic of static (**a**) and dynamic (**b**) high-temperature wettability test and measurement of contact angle.

High-temperature static wettability experiments were performed in the fouling test system (Figure 1a). The system included a special electric furnace and a set of high-speed cameras. It could be used to measure the static wettability and observe the dynamic process of impact and adhesion of molten corrosive fouling (MCF) on the surface of the specimen. According to the characteristics of the incineration fly ash, the corrosive fouling reagents in Table 2 were prepared by comprehensively considering the components of the corrosive and low-melting mixture, the deposition and slagging mixture, and the heavy metal mixture. The corrosive fouling reagent weighing 1 g was pressed into a cylindrical block and placed on the surface of the specimen (the mixed reagents all melted into a liquid state at 550 °C), and then put into an electric furnace to rapidly heat up to the experimental temperature (700, 750, 800, 850, or 900 °C). This process simulated how the MCF formed by fly ash in the boiler adheres to the wall. The melting process of fly ash on the specimen surface was photographed with a high-speed camera, and the morphological characteristics of the adhered surface after the MCF cooled were recorded.

In the dynamic high-temperature wettability experiment, we first tilted the 6 × 5 × 0.2 cm specimen by 45° and then heated it to the experimental temperature. Then, we dropped 1 g of MFC at the experimental temperature on the specimen surface at the dropping position, as shown in Figure 1b, and fixed the dropping position of all experiments. The dynamic wetting process of the MFC was captured by a high-speed camera, and the time from contacting the surface to sliding off the boundary was recorded (*L* no longer changed). We then cooled the sample, measured the length *L* of contact with the edge length after MFC left the surface, and took the average value three times.

The cross-section and surface morphologies were examined using SEM and EDX using a Hitachi Regulus 8100 (8100, HITACHI, Tokyo, Japan) equipped with an EDX system.

## 3. Results and Discussion

### 3.1. Coating Morphology

**Figure 2:**
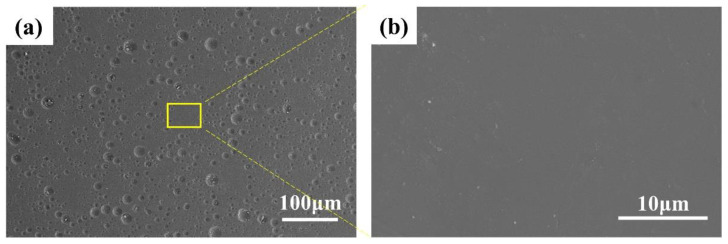
(**a**,**b**) SEM images of the coating surface.

Figure 2 shows the micromorphology and element distribution of the ceramic coating. The coating had a very dense outer surface that was smooth and uniform without micron-scale cracks and defects. The porosity of the coating, calculated by ImageJ (Version 1.8.0, National Institutes of Health, Bethesda, MD, USA) software, was less than 1%. The dense and smooth surface could effectively shield the penetration of MCF, making it difficult to form a corrosion core, and inhibiting the corrosion and scaling of the heated surface. Previous studies showed that the dense surface plays an important role in the practical application performance of the coating [15].

**Figure 3:**
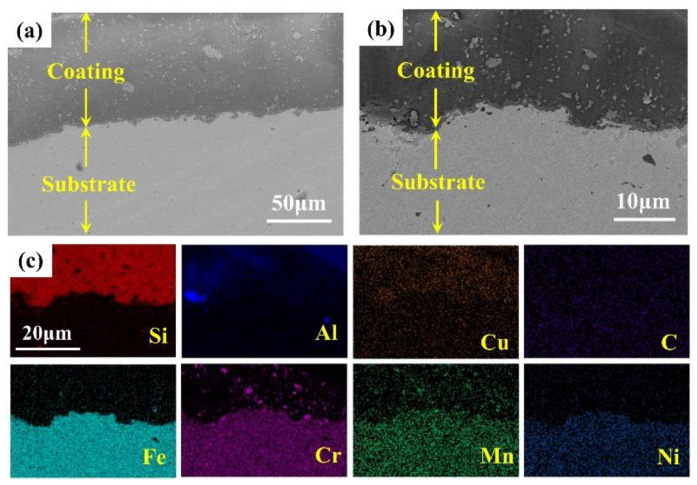
(**a**,**b**) SEM images of the cross-section; (**c**) EDX element spectrum of (**b**).

Figure 3 shows the micromorphology and element distribution of the cross-section. The coating mainly contained elements such as silicon, aluminum, and copper, and there was also penetration of metal elements. The benefit of the rational use of sodium silicate is that there were no micron-scale defects at the interface, forming an excellent bond with the substrate. The cross-section was very dense compared with what is reported in the literature [10,16,17].

### 3.2. Mechanical Properties

**Figure 4:**
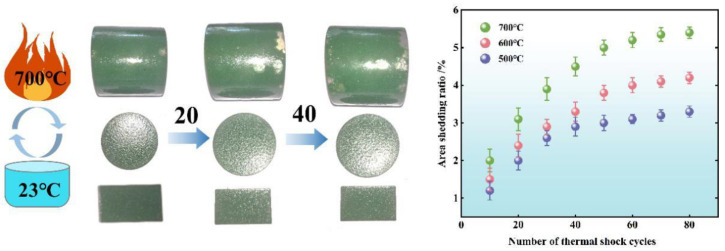
Macroscopic topography of thermal shock at 700 °C and peeling area ratio at different temperatures.

The mechanical properties of coatings are determined by thermal shock, which is caused by irregular fluctuations in the thermal load and abrasion wall, which are caused by rapid flue gas scouring in waste incinerators. According to the preparation method described in Section 2.2, the coatings were prepared on surfaces of three shapes made of SUS304 metal, namely circular tubes (φ30 × 6 × 30 mm), circular plates (φ30 × 2 mm), and rectangular flakes (25 × 15 × 2 mm). The thermal shock test was performed on the specimens after sintering in a muffle furnace. Figure 4 shows the macroscopic morphology of the three coated specimens after the 20th and 40th thermal shock at 700 °C. After 20 cycles of thermal shock, the coating did not appear to be noticeably peeling, and after 40 times, there was a small area of porcelain collapse in the edge area of the sample, and almost no peeling occurred after 50 thermal shocks. After 60 cycles, the peeling area was finally greater than 5%, which showed that the coating has good thermal shock resistance. The analysis of the damaged area showed that the edge of the coating is the area with the most concentrated stress and the most prominent position of the thermal expansion and contraction of the substrate. When these two positions overlap, the coating with poor forming quality in that area will be damaged.

**Figure 5:**
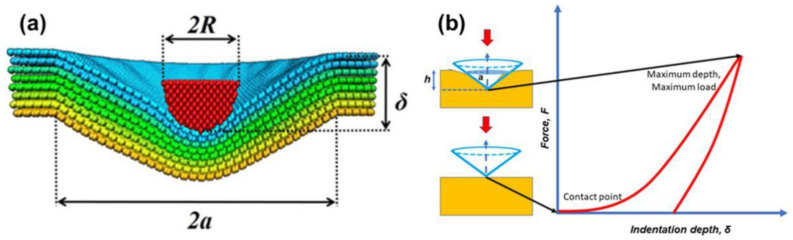
(**a**) The structural schematic of scratch theory. (**b**) The force vs. scratch curve [18].

**Figure 6:**
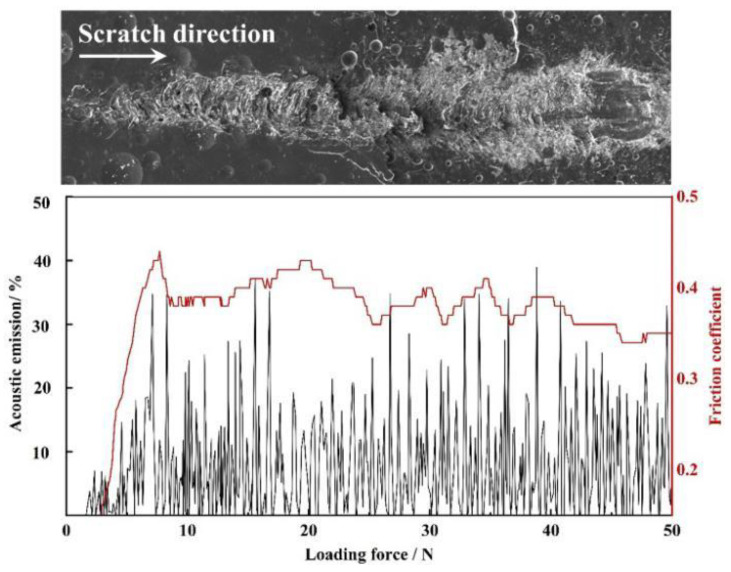
SEM and signal image of coating microscratch test.

Figure 5 is a schematic diagram of the theoretical structure of the microscratch experiment [18]; cohesive or adhesion failure of hard ceramic coatings may occur under the linearly increasing pressure load of the diamond stylus. Figure 6 shows the SEM and signal diagram of the coating scratch. The compressive stress damaged the surface of the coating, but there was no separation or complete separation from the substrate due to cracking and lifting, so we judged that cohesive failure had occurred. Combined with the signal diagram, we found the critical pressure load of the coating microscratch test was 7.13 ± 1.03 N, and the fundamental adhesion force was 25.14 ± 2.21 MPa.

**Figure 7:**
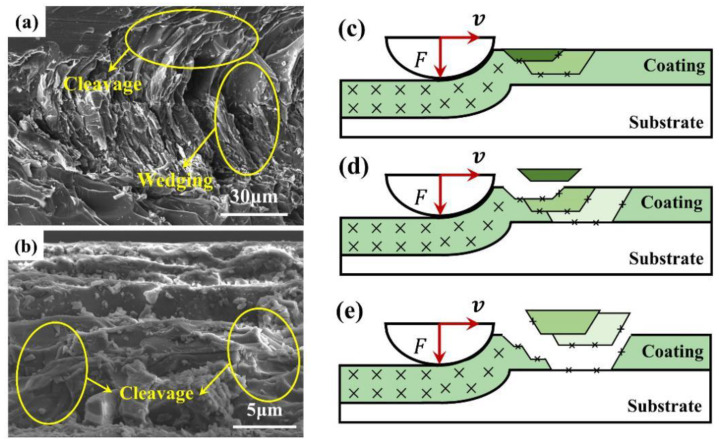
SEM images of the surface (**a**) and cross-section (**b**) of the coating. (**c**–**e**) Failure mechanism diagram.

Figure 7a,b show the SEM images of the surface and cross-section of the loaded area of the ceramic coating. The coating exhibited a regular morphology of cracking from top to bottom under the action of linearly increasing compressive stress, and the number and depth of cracked layers positively correlated. Combined with the analysis of the above adhesion test results, we found the coating exhibited a wedging failure mode and had a strong bonding strength. The reason for this morphology is that when the stylus exerts a load to a certain value, the coating has higher hardness and brittleness than the metal substrate, and shear cracks are generated inside the coating under the action of compressive stress. Then, further action causes the deformation difference and failure propagation of the coating on both sides of the shear crack. However, due to the strong bonding strength with the substrate, it could not be entirely separated, and finally resulted in a wedging failure mode morphology with cohesive failure. The mechanism is shown in Figure 7c–e.

The failure mode of the coating had a limited influence on the overall separation that may occur after pressure loading. From the material point of view, the coating may reasonably use mica powder. Based on previous studies, the mica phase has a tendency to undergo plastic flow and deformation under loading, and the interlocking structure in the ceramic also helps to effectively stop the cracks, thereby enhancing the plastic deformation of the material and decreasing the hardness [19,20]. The cleavage of the mica phase can be observed in Figure 7a,b. This mechanism is essential for applying the coating in incineration boilers. Compared with enamel coating, it has a much wider scope of application, as the coating can be sintered on prefabricated parts and then simply processed at a construction site.

### 3.3. High-Temperature Wetting Performance

**Figure 8:**
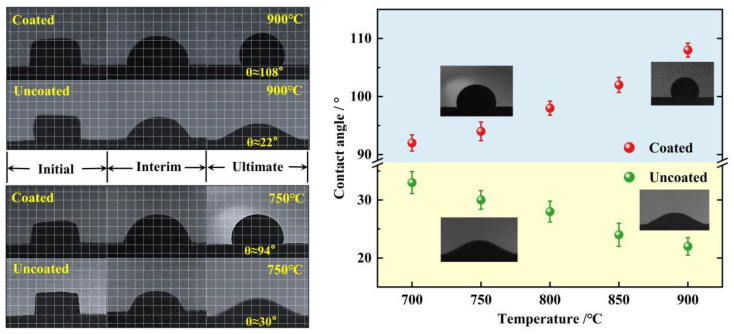
Contact angle changes and processes of MCF at different temperatures on coated and uncoated surfaces.

Figure 8 shows the morphology of MCF on the surface in the high-temperature static wettability experiment. As solid corrosives gradually melted by heating, a higher contact angle formed on the coated surface, showing a lyophobic character, and a lower contact angle formed on the uncoated steel surface, showing a lyophilic character. The higher the temperature, the greater the contact angle of coating surface, about 108° at 900 °C, and the smaller the contact angle of metal steel surface, about 22° at 900 °C.

**Figure 9:**
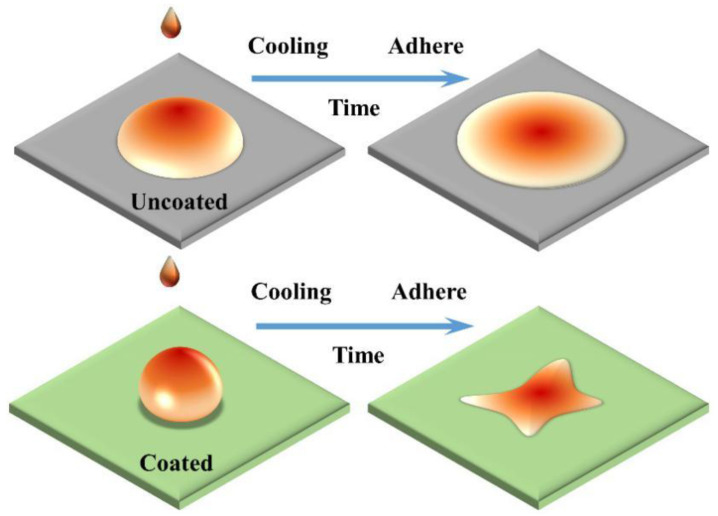
Schematic illustration of MCF adhesion after surface cooling.

Figure 9 is a conceptual schematic diagram of the MCF after cooling. The MCF on the uncoated surface exhibited a circular uniform spreading and adhesion, and the coated surface exhibited an irregular shape with irregular contact lines and small area shrinkage. According to a previous study, the smaller the contact angle, the stronger the adhesion to the MCF [21]. The strong adhesion is mainly manifested by the chemical reaction between the MCF and the surface to bond or adhere to a larger area. In experiments, we found that the uncoated steel surface was chemically bonded and adhered to a larger area. In comparison, the coating appeared to adhere to a smaller area without being chemically bonded, lowering the liquid-bridge force between the coating and MCF.

**Figure 10:**
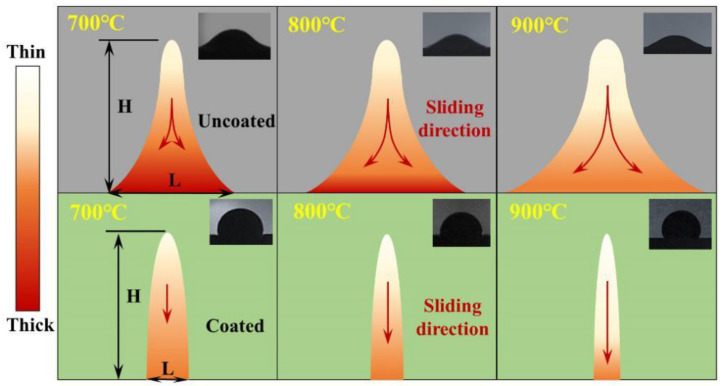
Qualitative schematic diagram of sliding traces on coated and uncoated surfaces after cooling in dynamic wettability test (the red arrows is the MCF sliding direction).

Figure 10 shows a qualitative schematic diagram of sliding traces after cooling of the coated and uncoated surfaces in the dynamic wettability test. From the figure, under the same sliding direction length, the MCF residue on the uncoated surface was thicker and sliding distance was longer, the contact line *L* with the surface boundary was longer, and the MCF spread and adhered to a larger area. Due to the low liquid-bridge force on the coating surface, it quickly slipped off the coating under the action of gravity, so there was no spreading or wetting of the surface, the MCF residue was thin, and the contact line *L* was very short.

**Figure 11:**
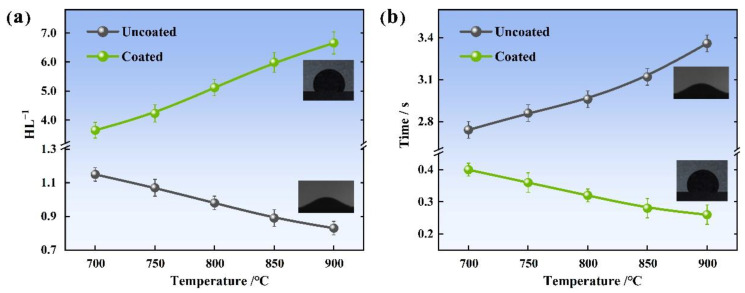
*HL*^−1^ and sliding time of MCF at different temperatures. (**a**) *HL*^−1^ varies with different temperatures; (**b**) Time varies with different temperatures.

*HL* is defined as the relative adhesion area: the larger the value, the larger the adhesion area. Figure 11a illustrates the *HL*^−1^ of the MCF on coated and uncoated surfaces at different temperatures. As can be seen, the *HL*^−1^ of the coated surface was larger than that of the uncoated surface at the experimental temperature: both increased with the increase in their respective contact angles. Figure 11b shows the sliding time of MCF on different surfaces. The sliding time on the coated surface was much shorter than that on the uncoated steel surface, and both decreased with the increase in their respective contact angles.

Many studies have shown that to achieve similar experimental phenomena at room temperature [22], the surface is usually transformed into a superhydrophobic surface with micro-/nano-layered textured structures or low surface energy [23]. Generally, as long as one of the two conditions is satisfied, the hydrophobic angle of the coating can reach more than 100° [24]. The surface morphology analysis in Section 3.1 shows that the coating has a dense and smooth outer surface on the glaze layer, the outermost layer is mainly composed of silicon, and more corrosion-resistant metal elements are deposited on the bottom layer. Therefore, we judged that the coating is lyophobic to MCF due to its lower surface energy. This indicated that the hydrophobic conditions at room temperature and the lyophobic conditions at high temperature are similar, because the condition characteristics and experimental results are similar.

Based on the analysis of the above experimental results, we found that the coated surface showed a lower contact angle and, therefore, a lower liquid-bridge force, compared with the uncoated steel surface. The antiscaling performance is mainly manifested in two aspects: First, the adhesion area is smaller, which is specifically evidenced by the MCF only concentrating in some areas rather than the entire heating surface in the boiler. Second, the shedding time is faster, and the thickness is smaller, which is manifested in the boiler, where the molten bottom layer cannot support many flue gas particles, resulting in the solid slag layer quickly falling off and re-exposing the coating surface instead of long-term accumulation occurring. These two aspects work together and do not exist in isolation. These characteristics mean that the ceramic coating has better antiscaling performance.

For applications in waste incineration boilers, the antifouling mechanism of the ceramic coating was described in combination with the above characteristic analysis as follows. At first, the low-melting fly ash and melt are adsorbed, or the flue gas hits the heating surface to form a thin viscous molten sublayer. Due to the coating surface’s low liquid-bridge force characteristics, the viscous molten bottom layer can only adsorb and deposit fewer insoluble flue gas large particles. When large particles accumulate to a certain amount after a certain period of time, they fall off together with the molten layer due to gravity and flue gas scouring in a relatively short period of time. These particles then re-expose the coated surface and quickly enter the next scaling cycle, which has a potential self-cleaning function. Due to the faster falling-off time and less deposition of fouling on the heating surface, the high-temperature corrosion rate is slowed, and the average fouling thermal resistance during the fouling cycle reduces, which enhances the heat transfer performance. Therefore, the two long-standing problems of the heating surface, fouling thermal resistance and corrosion, are alleviated.

**Figure 12:**
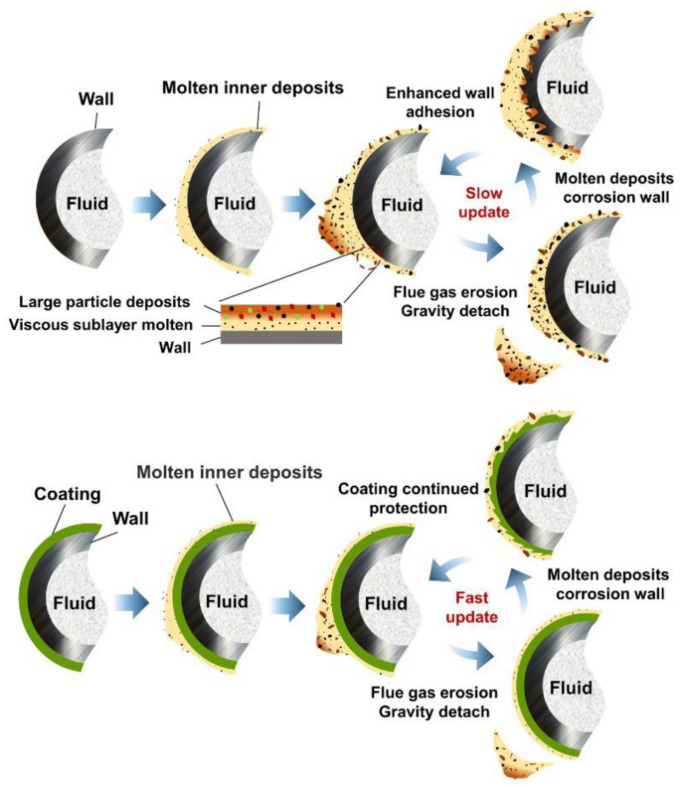
Working principle diagram of coated and uncoated steel in waste incinerator.

Compared with the coated surface, MCF usually does not easily and entirely fall off an uncoated steel surface, and a thicker solid slag layer remains. The slag layer slowly grows to form a tough “rock” that cannot fall off; finally, the heating surface can only be maintained by traditional mechanical descaling methods. The working principle is shown in Figure 12.

## 4. Conclusions

In this study, ceramic coatings were prepared on the surface of boiler steel by the slurry method. The coating morphology, interfacial bonding characteristics, applied mechanical properties, and antifouling properties were studied, and the following conclusions were obtained:

(1) Ceramic coatings can be prepared by the slurry method and in an environment simulating a start-up boiler. They can be sintered at a low temperature of 750 °C. The surface and cross-section are dense, the porosity is less than 1%, and the bonding strength is 25.14 ± 2.21 MPa.
(2) The thermal shock test showed that the coating peeled off about 5% of the coating after 60 cycles at 700 °C. The results of the microscratch test showed that the coating is not be completely separated after being subjected to pressure loading. The failure mode is wedging spalling due to cohesive failure, and coated products are machinable. The mechanical property test showed that the coating meets the primary requirements of practical applications.
(3) The high-temperature wettability experiment results showed that the ceramic coating has a smaller liquid-bridge force, smaller adhesion area, shorter fouling cycle for molten corrosive fouling, and has good antiscaling properties, and the potential for self-cleaning.
(4) The hydrophobic theory applied to normal temperature still plays a guiding role in studying the wettability of complex liquids in high-temperature environments.

## Figures and Tables

**Table 1:** Chemical composition of the slurry.

Raw Material	Mica	Copper	Chromium Oxide	Graphite	Sodium Silicate	Water	Dispersant	Defoamer
**wt.%**	**10**	**5**	**5**	**3**	**65**	**10**	**1**	**1**

**Table 2:** Composition of the corrosive mixture for high-temperature wettability test.

Corrosive Mixture	NaCl	KCl	Na_2_SO_4_	K_2_SO_4_	PbO	ZnO
**wt.%**	**20**	**20**	**20**	**20**	**10**	**10**

## Data Availability

The data presented in this study are available on request from the corresponding author.
